# Seroprevalence of Typhoid Fever and Its Associated Risk Factors Among Clinically Diagnosed Febrile Patients Visiting the Outpatient Department at Debark Hospital and Drug Susceptibility Patterns of Isolates

**DOI:** 10.1155/bmri/1717780

**Published:** 2025-02-01

**Authors:** Atsede Muleta, Naod Meseret

**Affiliations:** Department of Biology, College of Natural and Computational Sciences, University of Gondar, Gondar, Ethiopia

## Abstract

Typhoid fever is caused by the bacterium *Salmonella* Typhi, which poses major health problems in developing countries, including Ethiopia. However, there is limited information regarding typhoid fever, contributing factors with it, and its drug susceptibility pattern in the research area. The aim of this study was to determine the seroprevalence of typhoid fever and its associated risk factors among clinically diagnosed febrile patients at Debark Hospital and evaluate the drug susceptibility patterns of the isolates. A hospital-based cross-sectional study was conducted among 158 febrile patients from December 2022 to April 2023. Blood and stool samples were collected from each febrile patient. The Widal test was used to test *Salmonella* Typhi O and H antigens sera from blood, and bacteria were cultured from the stool. Gram staining and biochemical analyses were carried out for each isolate. Antibiotic susceptibility testing was performed for the isolates using Kirby–Bauer disk diffusion techniques. Descriptive statistics and logistic regression were used for analysis. In this study, the seroprevalence of typhoid fever among febrile patients at Debark Hospital was 22.8%. Gender (adjusted odds ratio (AOR): 95% confidence interval (CI): 0.02, 0.31, *p* ≤ 0.001), marital status (AOR: 95% CI: 2.63, 4.66, *p* ≤ 0.001), family size (AOR: 95% CI: 0.01, 0.31, *p* ≤ 0.001), residence (AOR: 95% CI: 0.09, 0.83, *p* = 0.021), practice of using toilet (AOR: 95% CI: 0.08, 0.086, *p* = 0.027), washing fruits and vegetables before eating (AOR: 95% CI: 0.12, 0.87, *p* = 0.025), and awareness of typhoid fever transmission and prevention (AOR: 95% CI: 0.12, 0.91, *p* = 0.032) are the associated risk factors for typhoid fever. Fifty percent of the isolates showed multidrug resistance to two or more antibiotics. It was suggested that improving personal hygiene, providing safe drinking water, and careful use of antibiotics could considerably reduce the prevalence of typhoid fever in the study area.

## 1. Introduction

Enteric fever is a systemic bacterial infection caused by *Salmonella enterica* serovars Typhi (*S.* Typhi) and *Salmonella enterica* serovars Paratyphi (*S.* Paratyphi) [1]. According to estimates, typhoid and paratyphoid fever affected 22 million people worldwide. Typhoid fever causes about 11–20 million cases and 210,000 deaths, and paratyphoid fever causes 5.4 million cases and 54,000 deaths every year worldwide [2]. Developing nations share the greatest burden due to rapid population growth, increased urbanization, and limited safe water and health system. The prevalence of this disease ranges from 160 per 100,000 people in sub-Saharan Africa to 0.3 per 100,000 people in Europe [3].

According to multicenter population-based surveillance, *Salmonella* Typhi was the most frequently isolated isolate of *Salmonella* spp. (24%), accounting for over 33% of all bacterial pathogen infections in Salmonella Shigella Agar medium [4]. Different findings showed that febrile patients had the highest rate of typhoid fever [3–5]. The fatality rate of typhoid fever cases ranges from 10% to 30% in the absence of treatment; however, rates can drop to 1%–4% with the right medication [2].

The bacteria are mostly transmitted by the fecal–oral pathway, which is consumed through raw milk products, flavored drinks, ice cream, and contaminated food and water. In low- and middle-income nations where sufficient diagnostic resources are unavailable, febrile illness symptoms are frequently misinterpreted [6]. The nonspecific clinical presentation of the disease, which includes fever, chills, dull frontal headache, malaise, anorexia, poorly localized abdominal discomfort, dry cough and myalgia, nausea, vomiting, constipation, and diarrhea, makes it difficult to distinguish it from other causes of fever, such as malaria, dengue, chikungunya, leptospirosis, scrub typhus, and influenza [7]. Other factors that contribute to this problem include a lack of resources, poor access to health facilities, and a shortage of medical professionals [8].

The evaluation of the burden of typhoid fever in endemic areas is limited to rapid serological tests, which have a low degree of sensitivity and specificity, making confirmed cases of typhoid fever unreliable [9]. Countries with an endemic incidence of typhoid fever lack well-established population-based national surveillance systems that created a substantial knowledge gap to inform policy and impact healthcare practice [8].

Typhoid fever is typically diagnosed in developing countries using clinical evidence and the Widal test results. The Widal test is comparatively less expensive, is simple to use, and needs little in the way of equipment or training. The Widal test is prone to error in diagnosing typhoid fever, with an overestimated 33% high prevalence of *S.* Typhi compared to a low culture-based pooled prevalence of 3% in Ethiopia [10]. This is despite the fact that the Widal slide agglutination test commonly used test the presence of antibody production against enteric fever [11]. Several factors bear significant risk for enteric fever, such as host characteristics, environmental exposures, and geographic and climatic factors [7].

Due to the lack of clarity around the diagnosis of *S.* Typhi, antibiotics are prescribed unnecessarily, which causes antibiotic resistance [12]. The emergence of antimicrobial resistance (AMR), especially multidrug resistance (MDR) to ampicillin, chloramphenicol, and cotrimoxazole, is a major public health problem that further complicates the treatment and management of typhoid fever [13]. The increase in AMR has been attributed to self-medication, inappropriate antibiotic usage, and a lack of clinical diagnostic tools to enable drug de-escalation in low- and middle-income countries [14, 15]. The clinical assessment of whether patients require antibacterial drugs and are amenable to empirical antibacterial therapy has been identified as one of the difficulties for clinicians in Ethiopia [10, 16].

Although Ethiopia lacks national surveillance studies on enteric fever, a study in central Ethiopia revealed a 4.1% prevalence of typhoid fever among febrile patients [17]. Furthermore, a systematic and meta-analysis study in Ethiopia showed that *S.* Typhi accounted for 42.1% of the total isolates of *Salmonella* species isolates, indicating that typhoid fever was endemic in Ethiopia [6, 17, 18]. However, data on the current prevalence, associated factors, and drug susceptibility patterns of enteric fever in the study area are limited. Also, Debark Hospital serves various districts, including Dabat, Janamora, Beyeda, Telemt, and Adiarkay; a large number of patients seek diagnosis there. Understanding area-specific monitoring studies helps to gain knowledge about rates, the types of pathogens, and their resistance patterns, which may help the clinician choose the correct empirical treatment. Therefore, the aim of this study was to determine the seroprevalence and associated factors of typhoid fever and the drug susceptibility pattern of the isolates among clinically diagnosed febrile patients who visited the outpatient department at Debark Hospital.

## 2. Materials and Methods

### 2.1. Description of the Study Area

The study was carried out in Debark Town, northwest Gondar Zone, Amhara Regional State, Ethiopia. Debark is situated at about 100, 280, and 841 km away from Gondar, Bahir Dar, and Addis Ababa, respectively. The town is geographically located at latitude and longitude of 13°08⁣′00⁣^″^ N and 37°54⁣′00⁣^″^ E, respectively, with an elevation of 2850 m above sea level. Debark town is the closest town to the Simien Mountains National Park and borders on the south by Dabat, on the west by Tegede, on the north by Adirkay, and on the east by Janamora, with an area of 1461.18 km^2^. According to the Central Statistics Agency of Ethiopia, the total population of the city is expected to be 227,526 of which 119,887 are males and 107,639 are females [19]. Most of the inhabitants practiced Ethiopian orthodox Christianity with 94.8%, while 5.2% of the inhabitants practiced Islamic religion. Regarding the health service status, the town has one health center and one hospital. This hospital was established in 1997 Gregorian calendar (GC). It is a referral center for districts in the area. It has a variety of specialists, including pediatricians, surgeons, gynecologists, and psychiatrists.

### 2.2. Study Design and Period

A hospital-based cross-sectional study was conducted from December 2022 to April 2023, at Debark Hospital, Debark, Ethiopia.

### 2.3. Study Population

The study population comprised febrile patients at Debark Hospital suspected of typhoid fever in the outpatient department (patients were recruited into the study regardless of their age) during the study period.

### 2.4. Sample Size Determination

The sample size estimation for the study was based on the previous typhoid fever prevalence study [20]. Employing assumptions of expected prevalence = 11%, the margin of error = 5%, *α* = 5% (95% confidence level), design effect = 2, and 5% nonresponse rate, a sample size of 158 enteric fever suspected patients were included in the study by using the formula for estimating single proportion [21].

### 2.5. Sampling Technique

The systematic random sampling method was used to recruit patients attending Debark Hospital. The daily total number of febrile patients who visited the hospital during the study period was divided by the sample size to determine the sample interval (*k*-value). The first patient was selected using the lottery method, and all patients of *K*th (where *k* = N/n, where k is the ratio of sampling frame size *N* and n is the desired sample size) were determined to participate in the study until the required sample size was obtained.

### 2.6. Inclusion and Exclusion Criteria

This study included febrile patients who had fever for two or more days before visiting Debark Hospital along with other clinical symptoms of typhoid fever and who were willing to participate after giving informed consent. Febrile patients who had received antibiotic treatment for their symptoms within 2 weeks prior to coming to Debark Hospital, those unable to consent to participate, and those who were diagnosed with other known febrile illnesses were excluded from the study.

### 2.7. Study Variables

The dependent variable of the study is the prevalence of typhoid fever in the study population. The independent variables were derived from sociodemographic and hygiene-related factors of the patient.

### 2.8. Data Collection Method

The data were collected using semistructured questionnaires via face-to-face interviews and laboratory diagnosis based on serological tests.

### 2.9. Specimen Collection and Processing

Blood and stool samples were taken from febrile patients for examination. The vein blood sample (2–3 mL) was collected aseptically using 70% alcohol in a sterile plain tube and centrifuged at 2500 rpm for 5 min to separate the serum. The fresh stool specimen provided by febrile patients was collected in a labeled screw cap container.

### 2.10. Laboratory Examination

#### 2.10.1. Serological Test

The serological test was carried out using a direct qualitative Widal slide agglutination test using *S.* Typhi O and H antigens according to the manufacturer's instructions. The antigen suspension commercially available in a 5 mL volume was used. In summary, the test was carried out by mixing one drop of serum with one drop of each O and H antigen separately on a slide. After the slide was swirled back and forth for 1 min, the mixture was observed for macroscopic agglutination. If there was agglutination within 1 min, it was reported as reactive for a positive result and nonreactive for a negative result.

#### 2.10.2. Bacterial Isolation and Identification

Approximately 1 g of stool sample was inoculated in selenite-cysteine broth contained in a sterile test tube and transported to the Medical Microbiology Laboratory, University of Gondar Referral Hospital, for culture. The samples were then incubated at room temperature overnight. A loop-full suspension is streaked on xylose–lysine–deoxycholate (XLD) agar and MacConkey agar at 37°C for 24 h for the isolation of *Salmonella*. The growth of isolates was detected by their characteristic appearance on XLD agar (red colonies with a black center) and on MacConkey agar (pale colonies). The colonies suspected to exhibit characteristics of *Salmonella* were further characterized by Gram staining and the following biochemical reaction tests, such as the catalase test, triple sugar iron agar, lysine iron agar, Simon's citrate agar, motility test, and urease test for identification [22].

#### 2.10.3. Drug Susceptibility Testing

The drug susceptibility test was performed using a Kirby–Bauer disk diffusion method [23]. Colonies from pure *Salmonella* cultures were transferred to a tube containing 5 mL of physiological sterile saline (0.85% NaCl). It was gently mixed until a homogeneous suspension formed. The suspension was adjusted equivalent to 0.5 McFarland standards and then uniformly lawn over the surface of Mueller–Hinton agar plates using a sterile cotton swab under a laminar hood. The inoculated plates were then left at room temperature to dry for 3–5 min; then, sterile forceps were used to lightly press the antibiotic disks manually on the surface of a Muller–Hinton plate to make a firm attachment. Accordingly, each isolate was subjected to seven antibiotic disks. The antibiotics used in this test were amoxicillin (10 *μ*g), ampicillin (10 *μ*g), chloramphenicol (30 *μ*g), ciprofloxacin (5 *μ*g), cotrimoxazole (5 *μ*g), gentamycin (10 *μ*g), and erythromycin (25 *μ*g) and incubated at 37°C for 18–24 h. The zone of inhibition was measured to the nearest millimeter, and the isolates were classified as sensitive (S), intermediate (I), or resistance (R) based on the interpretative criteria established by [24]. The isolates were also defined as resistant to multidrug if they were resistant to two or more tests of antibacterial agents.

### 2.11. Quality Control

As a means of quality control, the questionnaire designed to evaluate potential risk factors was pretested before the main trial on febrile patients. Trained laboratory technicians and nurses gathered the data. All samples were processed in accordance with standard operating procedures. Every morning, the appropriate operation of all the instruments used for processing samples was examined. All culture media were prepared following the manufacturer's instructions. A sample of culture medium plates prepared from each batch was incubated at 37°C for 24 h to check for sterility. Before inoculation, the culture media were visually inspected for contamination. Moreover, the McFarland standard was used to standardize the inoculum density of bacterial suspension for the antibiotic susceptibility test.

### 2.12. Data Analysis

Variables were defined, categorized, and coded. The data collected from the questionnaires and laboratory tests was entered into Microsoft Excel and analyzed using SPSS version 20. Sociodemographic characteristics were analyzed using descriptive statistics. Binary and multivariate logistic regression was calculated to examine the associated risk factors and prevalence of enteric fever. The first bivariate analysis was performed to select candidate variables for multivariate logistic regression analysis. Variables with a *p* value of 0.05 on bivariate analysis were selected for multivariate analysis. An odds ratio with their 95% confidence interval (CI) was used to determine the strength of association. *p* values less than 0.05 were considered statistically significant. Results were presented in the form of tables, figures, and descriptive statistics.

## 3. Results

### 3.1. Sociodemographic Characteristics of the Study Participants and Results of Serological Test

A total of 158 febrile patients who were suspected of having typhoid fever and visited Debark Hospital took part in the current investigation. Of the participants in the study, 92 (58.2%) were women. Regarding the study participants' ages, 53 (33.5%) of them belonged to the age group of 1–10 years, 44 (27.2%) to the age group of 11–20 years, and 10 (6.3%) to the age group of above 40. Of the total participants in the study, 38 (24.1%) and 37 (24.4%) completed secondary school and received diplomas, respectively. In terms of marital status, 91 (56.7%) of the study participants were single. The majority of study participants were employed as day workers 45 (28.5%), were orthodox 85 (53.8%), had more than six family members 78 (44.9%), and were urban dwellers 94 (59.5%) (Table 1).

**Figure 1:**
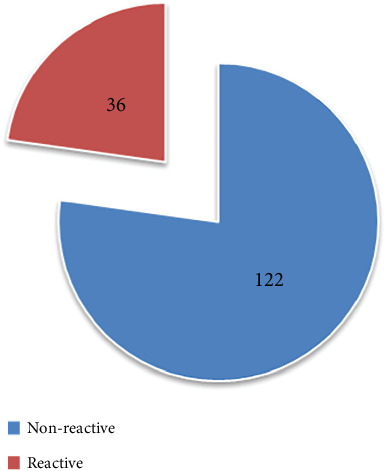
Widal slide agglutination test, *n* = 158.

Using *S.* Typhi O and H antigens, the direct qualitative Widal slide agglutination test produced the serological test result that showed that 122 (77%) of the 158 samples were nonreactive and 36 (22.8%) of the 158 samples were reactive (Figure 1).

### 3.2. Hygiene Practice of Typhoid Fever Patients

Table 2 illustrates that out of the study participants, 75 (47.5%) acquired their drinking water from the pipe, whereas 27 (17.1%), 46 (29.1%), and 10 (6.3%) obtained it from the well, spring, and river, respectively. One hundred fifteen (72.8%) of the 158 study participants did not treat their drinking water. Of them, 113 (75.5%) did not exhibit a tendency to use the toilet, and 121 (76.6%) did not exhibit a tendency to wash their hands after using the toilet. A total of 103 (65.2%) study participants were unaware of the transmission and prevention methods of typhoid fever, and 101 (63.9%) of them did not wash fruits and vegetables before eating.

### 3.3. The Seroprevalence of Typhoid Fever

In the current study, the total prevalence of patients with typhoid fever was 22.8% (Table 3). The majority of febrile patients were married, 26 (16.5%); illiterate, 10 (6.3%); women, 22 (13.89%); daily laborers, 11 (6.9%); orthodox, 23 (14.6%); and living in rural areas, 21 (13.8%). In terms of ages, 12 (7.6%) of those who had typhoid fever were between the ages of 1 and 10, and 11 (6.9%) were between the ages of 11 and 20. Twenty-seven (17.1%) of the patients with typhoid fever had more than six family members.

Regarding hygiene, most of the typhoid fever patients, 14 (8.9%), drank spring water, and 18.4% did not treat water before drinking. Almost all of them, 33 (20.9%), did not wash their hands after using the toilet, and 32 (20.3%) did not defecate in the toilet. The majority of 29 (18.4%) patients who had typhoid fever did not wash their fruits and vegetables before eating; 30 (19.0%) were unaware of the ways in which typhoid fever is transmitted and prevented (Table 4).

### 3.4. Associated Risk Factors for Typhoid Fever

As shown in Table 3, typhoid fever was significantly associated with gender, marital status, family size, and residence of febrile patients. However, there was no statistically significant association (*p* > 0.05) between typhoid fever and age, occupation, educational status, and religion.

With respect to the sex of febrile patients, women were 92% less likely to be infected with typhoid fever compared with men (adjusted odds ratio (AOR): 95% CI: 0.02, 0.31, *p* ≤ 0.001). The odds of typhoid fever seropositivity for married febrile patients were 11.99 times higher compared to those for unmarried (AOR: 95% CI: 2.63, 4.66, *p* ≤ 0.001). Concerning family size, those with fewer than three family members were 95% less likely to become infected with typhoid fever compared to those with more than six family members (AOR: 95% CI: 0.01, 0.31,*p* ≤ 0.001). Regarding the residence of febrile patients, urban dwellers were 71% less likely to become infected with typhoid fever compared to rural dwellers (AOR: 95% CI: 0.09, 0.83, *p* = 0.021).

The risk of typhoid fever infection was 2.53 times higher in those age groups between 1 and 10 years (AOR: 95% CI 0.06, 0.99, *p* = 0.581) than > 40 years. Similarly, the risk of typhoid fever infection was 1.29 times higher in age group 11–20 years compared to ≥ 40 years of age (AOR: 95% CI: 0.03, 0.85, and 0.293). The risk of typhoid fever infection was 4.89 times higher in illiterates than those who had degrees and above groups (95% CI 0.76, 1.45, *p* = 0.094). Those respondents who are daily laborers are 2.41 times more likely to get infected with typhoid fever than those who are students (AOR: 95% CI: 0.10, 1.71, *p* = 0.222). Orthodox followers of religion had a 15.29 chance of getting infected with typhoid fever compared to those with protestant religions (AOR: 95% CI: 1.00, 2.33, *p* = 0.050).

Typhoid fever was significantly correlated with the following hygiene habits, as shown in Table 4: using the toilet, washing hands after using the toilet, cleaning fruits and vegetables before eating, and being aware of the spread and prevention of typhoid fever. On the other hand, no statistically significant correlation (*p* > 0.05) was found between the source and treatment of drinking water and typhoid fever.

Febrile patients who used the toilets had a 74% lower risk of getting typhoid fever compared to those with their counterparts (AOR: 95% CI: 0.08, 0.86, *p* = 0.027). There was a 76% reduction in the risk of getting typhoid fever among study participants who washed their hands after using the toilet (AOR: 95% CI: 0.07, 0.91, *p* = 0.036). Those study participants who wash their fruits and vegetables before eating are 67% less likely to get typhoid fever than those who had the illness but did not wash their fruits and vegetables before eating (AOR: 95% CI: 0.12, 0.87, *p* = 0.025). Febrile patients who were aware of the transmission and prevention methods of typhoid fever had a 68% lower risk of developing typhoid fever than those who were not (AOR: 95% CI: 0.12, 0.91, *p* = 0.032).

The febrile patients who drank well and spring water were 1.05 (AOR: 95% CI: 0.18, 0.19, *p* = 0.949) and 1.22 (AOR: 95% CI: 0.23, 0.54, *p* = 0.813) at higher risk of getting infected with typhoid fever compared to those with who drink river water. Contrarily, compared to those with who drink river water, febrile patients who drink water from pipes had a 57% (AOR: 95% CI: 0.08, 0.21, *p* = 0.309) lower risk of getting infected with typhoid fever. Study participants who treated their drinking water before consumption were 48% less likely to become infected with typhoid fever compared to those with who did not treat their drinking water before consumption (AOR: 95% CI: 0.19, 1.41, *p* = 0.195).

### 3.5. Characterization of Bacterial Isolate From Stool Sample

A total of six bacteria were isolated from the stool sample (Table 5). The isolates grown in MacConkey agar medium have round pale colonies, and in XLD agar medium, they showed as red colonies with a black center. All isolates were Gram-negative, were capable of assimilating catalase, and showed negative urease activities with motile characteristics. Four isolates were positive for the triple sugar iron (TSI) and lysine iron agar (LIA) tests, whereas two isolates (ST3 and ST6) exhibited a negative result. However, two isolates (ST3 and ST6) were able to use citrate, while four isolates had negative results for citrate activity. Based on microscopic, cultural, and biochemical test results, all bacterial isolates were most likely classified into the genus *Salmonella* (Table 6). Four isolates were thought to be *S.* Typhi, and the other two might be *S.* Paratyphi.

### 3.6. Drug Susceptibility Patterns of *Salmonella* Isolates

The susceptibility pattern of *Salmonella* isolated from stool culture to seven antibiotics is presented in Table 6. Drug susceptibility testing revealed that approximately three (50%) isolates were susceptible to ciprofloxacin.

In the current study, all isolates showed resistance to ampicillin and erythromycin 6 (100%). Similarly, 4 (66.6%) of the isolates showed a high resistance rate to amoxicillin. However, some of the isolates were sensitive to chloramphenicol 2 (33.3%) and gentamicin and cotrimoxazole 1 (16.6%) (Table 6).

The three isolates (50%) showed multidrug drug resistance to ampicillin, amoxicillin, and erythromycin. Furthermore, an isolate exhibited multidrug resistance to almost all antibiotics except ciprofloxacin (Table 7).

## 4. Discussion

It was found that the seroprevalence of typhoid fever among febrile patients at Debark Hospital was 22.8%. This finding was much higher than the study conducted in central Ethiopia (4.1%) [17], Bahir Dar (5.3%) [25], and Indonesia (15.5%) [26]. It was lower than the report from West Wellega (53.6%) [27], Addis Ababa (51.39%) [18], Arerti (30%) [28] and Debre Birhan (69.23%) [29]. It is possible to explain this discrepancy by varying the sample size, the study area's geographic location, the seasonality of the study periods, and the laboratory investigation approach [6, 30].

Males were shown to be more strongly influenced by typhoid fever than females. This could be explained by the fact that men are usually the family's earners and spend a greater amount of time away from home, which raises the possibility that they will eat out and practice hygiene [31]. In terms of the age range of febrile patients, those between the ages of 1 and 10 and 21 and 30 had a greater incidence of typhoid fever. GBD and Habte et al. [2, 18] reported a comparable result. This may be due to the sanitary and feeding attitudes of these age groups [32]. According to Bhandari et al. [7], these age groups are representative when typhoid fever incidence rises. The prevalence of typhoid fever is linked to a lower peak age incidence; however, this masks notable geographical differences in the age of onset. Because of higher exposures and greater acquired immunity from recurrent clinical, subclinical, or silent infections as people age, peak incidence may occur in areas with exceptionally high frequency.

This study found a significant association between the prevalence of typhoid fever and marital status. Awol, Reda, and Gidebo reported an insignificant association between the prevalence of typhoid fever and marital status [33]. This discrepancy could be brought about by the study's design and geographical variations. In this study, married patients had a higher risk of typhoid fever than unmarried individuals. It is unclear why married patients have a higher risk of typhoid fever than single patients, but many of them are compelled by marital duties to prepare meals at home. Since contaminated food and water are key sources of typhoid fever infections, those who do not follow basic hygiene standards are also more likely to have typhoid fever [31].

Those study participants who had less than three family members were at a lower risk of getting infected with typhoid fever than those with more than six family members. Deksissa and Gebremedhin [11] reported that study participants with more than five family members were infected with typhoid fever at a higher rate than those with fewer family members. This could mean that food made by small family members is more likely to be fresh or hygienic than food made by a larger family. According to a USFDA [34] report, good hygiene during food handling cannot lead to the spread of *Salmonella* in foods.

Regarding the residence of the study participants, patients who live in the rural area were more infected with typhoid fever than those who live in urban areas. This concurs with the finding of Awol, Reda, and Gidebo [33], where patients living in rural areas had an eightfold higher risk of having typhoid fever compared to those with living in urban areas. The increased risk of typhoid fever in rural areas can be presumed to be due to a number of factors, such as poor personal hygiene, low socioeconomic status, open defecation near springs and rivers, lack of access to sanitary housing and clean water, absence of toilets and/or hand washing facilities after using the restroom, and lack of medical attention [18].

The prevalence of typhoid fever and the practice of using toilets are significantly associated. Febrile patients who used the toilets had a lower risk of getting typhoid fever. According to this result, Deksissa and Gebremedhin [11] confirmed that febrile patients who had toilet use practice were less likely to become infected with typhoid fever, and the association between variables was significant. Then again, Amsalu, Genet, and Adem Siraj [25] confirmed that febrile patients who had toilet use practice were less likely to get infected with typhoid fever, but an insignificant association between variables was observed. This is related to the fact that typhoid fever is primarily an intrahome issue, spread by a recent or active case in the family, and made easier by inadequate hand washing and personal hygiene [35].

In this study, washing fruits and vegetables before eating was significantly associated with the prevalence of typhoid fever infection. However, these were contradictory to a study conducted in Hawassa, Ethiopia [33], which found that washing fruits and vegetables before eating was not significantly associated with the prevalence of typhoid fever. This discrepancy may have resulted from geographical differences that influence different hygienic and food consumption practices [25]. Febrile patients who wash their fruits and vegetables before eating are less likely to get typhoid fever than those who had the illness but did not wash their fruits and vegetables before eating. This finding was comparable with a study done in Injibara General Hospital, Northwest Ethiopia [36]. This may be because eating fruits and vegetables that have been thoroughly cleaned before consumption lowers the risk of microorganisms causing a foodborne illness [37].

The transmission and prevention of typhoid fever awareness were found to be significantly associated with the prevalence of typhoid fever infection; additionally, febrile patients who were aware of these factors had a lower risk of developing typhoid fever than those who were not. Deksissa and Gebremedhin [11] reported that there was no significant association between the variables, but study participants who were unaware of the transmission of typhoid fever had a higher risk of getting infected with typhoid fever and a higher risk of using preventive methods. This could be explained by the fact that typhoid fever is less common in those who follow good hygiene and have adequate awareness or education [38].

According to the results of a drug sensitivity test, the *Salmonella* isolate was 100% resistant to ampicillin and erythromycin. This is in agreement with the findings of the previous studies reported in different areas [39, 40]. This could be caused by inadequate supplies, condensed antimicrobial therapy, bacterial development, temperature shifts, and low-quality drugs. Antibiotic resistance is mediated by genetic components that encode the mechanisms by which bacteria avoid the effects of antibiotics. *Salmonella* bacterium has a thin peptidoglycan layer followed by an outer membrane. Because of this, antibiotics targeting cell wall synthesis, such as ampicillin and other semisynthetic b-lactams, can stop it from growing by passing through porins in the outer membrane. Macrolides like erythromycin can be used as alternatives for b-lactam antibiotics to treat bacterial infections by inhibiting protein synthesis and translation [4].

In this study, 50% of *Salmonella* isolates showed susceptibility towards ciprofloxacin, which is similar to the earlier findings [32, 41]. One possible explanation for this could be the long-standing obsolescence of common antibiotics and the pathogenic strains' loss-resistant–inducing plasmids through mutation [42]. According to this study, 50% of *Salmonella* isolates were multidrug-resistant to two or more antibiotics. This was consistent with earlier research from Bangladesh and Kenya, which showed 68% and 85% MDR, respectively [43, 44]. This could indicate that patients taking those antimicrobial drugs had a higher risk of negative outcomes, a poor clinical response, and a severe illness with possible complications [42].

Even though it could report important findings, the current study had several limitations, including failure to determine the end titer of antibodies, the inability to culture from blood, the small sample size, no species identification performed for the *Salmonella* isolates, and failure to use all recommended drugs for antimicrobial susceptibility testing due to a shortage. Furthermore, it is important to exercise caution when extrapolating the results of a cross-sectional study conducted in a hospital to the broader population. Conversely, our data offer a foundation for population-based research to determine the typhoid fever burden.

## 5. Conclusions and Recommendations

In conclusion, the results revealed that the overall seroprevalence of typhoid fever among febrile patients in the study area was 22.8%. Sex, marital status, family size, residence of patients, toilet usage practice of patients, washing fruits and vegetables before eating, and awareness of typhoid fever transmission and prevention are the most significant associated risk factors for the prevalence of enteric fever in the study area. Three (50%) *Salmonella* isolates showed susceptibility to ciprofloxacin, but six (100%) were shown to be resistant to ampicillin and erythromycin. Most of the *Salmonella* isolates showed MDR to two or more antibiotics utilized in the current study. It was suggested that improving personal and environmental hygiene, providing safe drinking water, engaging in extensive health education, and using antimicrobials more carefully could preserve their effectiveness and considerably reduce the prevalence of typhoid fever in the study area.

## Figures and Tables

**Table 1:** Sociodemographic characteristics of typhoid fever patients visited the outpatient department of Debark General Hospital, from December 2022 to April 2023, *n* = 158.

**Variables**	**No.**	**Percentage (%)**
Sex		
Female	92	58.2
Male	66	41.8
Total	158	100%
Age		
1–10	53	33.5
11–20	44	27.8
21–30	29	18.4
31–40	22	13.9
> 40	10	6.3
Total	158	100%
Education		
Illiterate	33	20.9
Primary education	26	16.5
Secondary education	38	24.1
Diploma	37	23.4
Degree and above	24	15.2
Total	158	100%
Marital status		
Married	67	42.4
Unmarried	91	57.6
Total	158	100%
Occupation		
Merchant	24	15.2
Employed	17	10.8
Farmer	40	25.3
Daily laborer	45	28.5
Student	32	20.3
Total	158	100%
Family size		
<3 members	32	20.3
3–6 members	48	30.4
> 6 members	78	49.4
Total	158	100%
Religion		
Orthodox	85	53.8
Muslim	62	39.2
Others	11	7.0
Total	158	100%
Residence		
Urban	94	59.5
Rural	64	40.5
Total	158	100%

**Table 2:** Hygiene practice of typhoid fever patients who visited the outpatient department of Debark General Hospital, from December 2022 to April 2023, *n* = 158.

**Variables**	**No.**	**Percentage (%)**
Drinking water source		
Pipe	75	47.5
Well	27	17.1
Spring	46	29.1
River	10	6.3
Total	158	100%
Treating drinking water		
Yes	43	27.2
No	115	72.8
Total	158	100%
Toilet usage		
Yes	45	28.5
No	113	75.5
Total	158	100%
Hand washing after toilet usage		
Yes	37	23.4
No	121	76.6
Total	158	100%
Washing fruits and vegetables before eating		
Yes	57	36.1
No	101	63.9
Total	158	100%
Awareness of typhoid fever transmission and prevention		
Yes	55	34.8
No	103	65.2
Total	158	100%

**Table 3:** Binary logistic regression analysis (COR) and multivariate logistic regression analysis (AOR) for associated risk factors with seroprevalence of typhoid fever among febrile patients who visited the outpatient department of Debark General Hospital, from December 2022 to April 2023, *n* = 158.

**Variables**	**No. of tested febrile patients**	**No. of positive patients**	**Percentage (%)**	**COR (95% CI)**	**p** ** value**	**AOR (95% CI)**	**p** ** value**
Sex							
Male	92	26	16.5	1		1	
Female	66	10	6.3	2.44 (1.09, 5.48)⁣^∗^	0.032	0.08 (0.02, 0.31)⁣^∗∗^	0.001
Age							
1–10	53	12	7.6	1.85 (0.16, 0.57)	0.854	2.53 (0.06, 0.99)	0.581
11–20	44	11	6.9	2.75 (0.14, 0.48)	0.709	1.29 (0.03, 0.85)	0.293
21–30	29	8	5.1	0.66 (0.11, 0.78)	0.637	0.54 (0.05, 0.68)	0.606
31–40	22	3	1.9	0.58 (0.22, 0.36)	0.648	0.14 (0.01, 1.94)	0.141
> 40	10	2	1.3	1		1	
Education							
Illiterate	33	10	6.3	0.33 (0.08, 1.36)	0.124	4.89 (0.76, 1.45)	0.094
Primary	26	6	3.8	0.48 (0.11, 1.17)	0.337	1.05 (0.14, 0.92)	0.965
Secondary	38	8	5.1	0.54 (0.13, 1.26)	0.395	4.61 (0.79, 1.26)	0.086
Diploma	37	9	5.7	0.44 (0.51, 1.85)	0.264	2.85 (0.40, 1.09)	0.294
Degree and above	24	3	1.9	1		1	
Marital status							
Married	67	22	13.9	0.37 (0.17, 0.79)⁣^∗^	0.011	11.99 (2.63, 4.66)⁣^∗∗^	0.001
Unmarried	91	14	8.9	1		1	
Occupation							
Merchant	24	6	3.8	1.53 (0.29, 3.39)	0.149	0.28 (0.05, 1.57)	0.149
Employed	17	2	1.3	1.50 (0.47, 1.39)	0.285	0.15 (0.02, 1.32)	0.087
Farmer	40	9	5.7	1.15 (0.39, 3.42)	0.804	1.41 (0.09, 1.79)	0.239
Daily laborer	45	11	6.9	1.03 (0.36, 2.94)	0.956	2.41 (0.10, 1.71)	0.222
Student	32	8	5.1	1		1	
Family size							
< 3	32	2	1.3	7.94 (1.76, 35.78)⁣^∗^	0.007	0.05 (0.01, 0.31)⁣^∗∗^	0.001
3–6	48	7	4.4	3.10 (1.23, 7.84)	0.017	0.29 (0.08, 1.09)	0.067
> 6	78	27	17.1	1		1	
Religion							
Orthodox	85	23	14.6	0.57 (0.03, 2.23)	0.223	15.29 (1.00, 2.33)	0.050
Muslim	62	12	7.6	0.42 (0.05, 3.58)	0.425	11.19 (0.68, 1.89)	0.091
Protestant	11	1	0.6	1		1	
Residence							
Urban	94	15	9.5	2.57 (1.20, 5.49)⁣^∗^	0.015	0.29 (0.09, 0.83)⁣^∗^	0.021
Rural	64	21	13.3	1		1	

Abbreviations: AOR, adjusted odds ratio; CI, confidence interval; COR, crude odds ratio.

⁣^∗^*p* value < 0.05.

⁣^∗∗^*p* value < 0.01: significant association.

**Table 4:** Binary logistic regression analysis (COR) and multivariate logistic regression analysis (AOR) for hygiene practice and its association with typhoid fever among febrile patients who visited outpatient department of Debark General Hospital, from December 2022 to April 2023, *n* = 158.

**Variables**	**No. of tested febrile patients**	**No. of positive patients**	**Percentage (%)**	**COR (95% CI)**	**p** ** value**	**AOR (95% CI)**	**p** ** value**
Drinking water source							
Pipe	75	11	7.0	2.49 (0.56, 1.13)	0.231	0.43 (0.08, 0.21)	0.309
Well	27	8	5.0	1.02 (0.21, 4.97)	0.983	1.05 (0.18, 0.19)	0.949
Spring	46	14	8.9	0.98 (0.22, 4.35)	0.978	1.22 (0.23, 0.54)	0.813
River	10	3	1.9	1		1	
Treating drinking water							
Yes	43	7	4.4	1.73 (0.69, 4.32)	0.237	0.52 (0.19, 0.41)	0.195
No	115	29	18.4	1		1	
Toilet usage							
Yes	45	4	2.5	4.05 (2.33, 2.23)⁣^∗^	0.013	0.26 (0.08, 0.86)⁣^∗^	0.027
No	113	32	20.3	1		1	
Hand washing after toilet usage							
Yes	37	3	1.9	4.25 (1.22, 1.38)⁣^∗^	0.023	0.24 (0.07, 0.91)⁣^∗^	0.036
No	121	33	20.9	1		1	
Washing fruits and vegetables before eating							
Yes	57	7	4.4	2.88 (1.17, 2.08)⁣^∗^	0.021	0.33 (0.12, 0.87)⁣^∗^	0.025
No	101	29	18.4	1		1	
Awareness of typhoid fever transmission and prevention							
Yes	55	6	3.8	3.36 (1.30, 8.67)⁣^∗^	0.012	0.32 (0.12, 0.91)⁣^∗^	0.032
No	103	30	19.0	1		1	

Abbreviations: AOR, adjusted odds ratio; CI, confidence interval; COR, crude odds ratio.

⁣^∗^*p* value < 0.05: significant association.

**Table 5:** Gram reaction test and biochemical characterization *Salmonella* isolates, *n* = 6.

**Codes of isolates**	**Gram test**	**TSI test**	**Catalase test**	**Urease test**	**Motility test**	**LIA test**	**Citrate test**	**Identified under genus**
ST1	−	+	+	−	Motile	+	−	*Salmonella*
ST2	−	+	+	−	Motile	+	−	*Salmonella*
ST3	−	—	+	−	Motile	−	+	*Salmonella*
ST4	−	+	+	−	Motile	+	−	*Salmonella*
ST5	−	+	+	−	Motile	+	−	*Salmonella*
ST6	−	−	+	−	Motile	−	+	*Salmonella*

Abbreviations: LIA: lysine iron agar; TSI: triple sugar iron agar.

**Table 6:** Drug susceptibility pattern of *Salmonella* isolates on the disk diffusion test isolated from patients attending Debark General Hospital, interpreted from the susceptibility test measure by CLSI (2011).

**Antibiotics**	**Antimicrobial susceptibility pattern**					
	**Susceptible**		**Intermediate**		**Resistance**	
	**Number of isolates**	**%**	**Number of isolates**	**%**	**Number of isolates**	**%**
Amoxicillin	—	—	2	33.3%	4	66.6%
Ampicillin	—	—	—	—	6	100%
Chloramphenicol	2	33.3%	3	50%	1	16.6%
Ciprofloxacin	3	50%	3	50%	—	—
Erythromycin	—	—	—	—	6	100%
Gentamycin	1	16.6%	4	66.6%	1	16.6%
Cotrimoxazole	1	16.6%	3	50%	2	33.3%

**Table 7:** Multidrug resistance pattern of *Salmonella* isolates on disk diffusion test isolated from patients attending Debark General Hospital.

**Resistant types**	**MDR pattern**	**No. of isolates**	**%MDR**
Amc, Amp, E, C, Cot, Gen	6	1	16.6%
Amc, Amp, E, Cot	4	2	33.3%
Amc, Amp, E	3	3	50%
Amp, E	2	6	100%

Abbreviations: Amc: amoxicillin; Amp: ampicillin; C: chloramphenicol; Cip: ciprofloxacin; Cot: cotrimoxazole; E: erythromycin; Gen: gentamycin.

## Data Availability

All data and materials supporting the final results are presented in the manuscript.
